# The effects of using an active workstation on executive function in Chinese college students

**DOI:** 10.1371/journal.pone.0197740

**Published:** 2018-06-07

**Authors:** Zhanjia Zhang, Bing Zhang, Chunmei Cao, Weiyun Chen

**Affiliations:** 1 School of Kinesiology, University of Michigan, Ann Arbor, Michigan, United States of America; 2 Department of Sports Science and Physical Education, Tsinghua University, Beijing, China; University of Illinois at Urbana-Champaign, UNITED STATES

## Abstract

This study aimed to examine the effects of active workstation use on the executive function by measuring the three components of executive function (Inhibition, Updating, and Shifting) during sitting, standing, and walking at an active workstation with different speeds. Twenty-four college students completed a cognitive test battery while sitting, standing, walking on an active workstation with a self-selected speed (mean = 2.3 km/h) and a faster speed (mean = 3.5 km/h). The three components of executive function (Inhibition, Updating, and Shifting) were assessed by Stroop task, N-back task, More-odd shifting task, respectively. Performance of each task was determined by the response time and accuracy. Repeated measures ANOVAs were conducted with workstation condition and trial type as within-subjects factors. There were no significant main effects for workstation condition and no interaction between workstation condition × trial type in Stroop task and More-odd shifting task. There was a significant main effect for workstation condition (F (3, 69) = 4.029, p = 0.011) and interaction effect between workstation condition × trial type (F (6, 138) = 9.371, p < 0.001) in N-back task. Decomposition of the interaction showed that accuracy of 2-back task in self-paced walking was significantly lower than that in sitting condition (p = 0.017) and in standing condition (p < .001). But there was no difference in accuracy of 2-back task between self-paced walking condition and faster walking condition (p = 0.517). Our results suggest that using an active workstation may have a selective impact on three components of executive function, in which the Updating may be impaired to a certain extent while the Inhibition and Shifting remain unaffected.

## Introduction

Sedentary lifestyle and physical inactivity have numerous adverse effects on health, such as increased morbidity of cardiovascular diseases and higher mortality from all causes [1]. Unfortunately, the opportunities for physical activity have been largely eliminated due to the changes in the way we work, commute, and spend leisure time [2]. It has been pointed out that the rapid development of technology in the past fifty years has been making our environment more inclined to result in physical inactivity and sedentary lifestyle [3]. According to a survey, the percentage of US adults occupied in sedentary work has increased by 67%, and 46% people spend almost all their work time sitting [3]. Since nearly half of our waking hours are spent at work, the sedentary work environment largely accounts for daily physical inactivity. At present, the major strategy to address the issue of physical inactivity due to the sedentary work environment is to encourage employees to engage in more exercise after work such as by providing some free fitness programs for employees or using financial incentives [4]. However, a systematic review showed that such worksite physical activity promotion strategies were not effective as they intended to be [5]. A study assessed the employee’s attitude toward worksite health promotion services and found that the main reported barriers to participating the worksite health programs included no time during the workday and no time before or after work [6]. In addition, the main reported incentives that would promote the employee’s physical activity include convenient time and convenient location. Given these barriers and incentives, it might be effective to promote physical activity if the way of doing physical activity does not occupy the time after work and occurs at convenient location, which was the very idea that the active workstation was derived from.

The idea of active workstation was first proposed by Edelson and Danoffz in 1989. Edelson and Danoffz [7] designed the active workstation by combining a treadmill and an office desk together so that people were able to perform working tasks while walking. Since the active workstation was designed to reduce sedentariness and promote physical activity at workplace, researchers were concerned about two questions in its application: 1) Does the use of the active workstation effectively increase physical activity at workplace? 2) Will the use of the active workstation significantly influence work performance? Results from previous studies consistently showed that the use of active workstation effectively improved physical activity (PA) and increased energy consumption at workplace. For instance, Levine and Miller [8] found that the energy consumption while walking at 1.1 mph at an active workstation was 191 kcal/h, which was 119 kcal/h higher than that in sitting condition. Regarding the influence of the use of active workstation on work conformance, results of the existing literature were inconsistent due to the large variability of participants and measurement. For example, some early studies adopted typing performance as outcomes reporting detrimental impact [9, 10] while some used subjective measurement such as supervisors’ rating showing no impairment [11]. More recently, a growing literature emerged focusing on the effects of active workstation use on cognitive functions. For instance, Ohlinger [12] administered Stroop Color Word test and Auditory Consonant Trigram test and showed that walking on an active workstation with 1 mph did not affect selective attention and short-term auditory verbal memory. Similarly, Alderman et al. [13] also revealed that selective attention was not affected when walking on an active workstation with self-selected speed. Ehhamn et al. [14] examined executive function and found the executive function performance was relatively unaffected while walking on an active workstation with self-selected speed. The effects of the use of active workstation on cognitive functions might be the potential foundation for work performance while using the active workstation. Therefore, investigations of the relationship between the use of active workstation and cognitive functions may help us to understand what types of working tasks may be influenced and what types may not, and thus to better facilitate the use the active workstation.

Among the cognitive functions, executive function is considered as a higher-order cognitive function since it matures the latest and controls lower-order cognitive functions [15]. Executive function is a set of cognitive processes that modulate and coordinate various cognitive processes to facilitate the attainment of chosen goals [16]. According to the theoretical model proposed by Miyake et al. [17], executive function consists of three core components——inhibition of proponent responses (“Inhibition”), information updating and monitoring (“Updating”), and mental set shifting (“Shifting”). The Inhibition refers to one’s ability to deliberately inhibit dominant, automatic, or prepotent responses when necessary [17]. The Updating, which is highly close to the notion of working memory, refers to one’s ability to monitor and code incoming information for relevance to the task at hand and replace old, no longer relevant information with newer, more relevant information [17]. The last component of executive function requires shifting back and forth between multiple tasks, operations, or mental sets [17]. Given the importance of the executive function, it was of our interest to investigate whether the use of active workstation has an impact on executive function. Additionally, since the executive function consists of three components that are related to different cognitive processes, we were also interested in the effects of the use of active workstation on each of the three components of executive function.

Taken together, as a promising way to promote physical activity workplace, the influences of the use of active workstation on cognitive functions have remained largely unexplored. The purpose of this study was to examine the effects of active workstation use on the three components of executive function. Specifically, we measured and compared the three components of executive function when people were sitting, standing, and walking on an active workstation with different speeds (one self-selected speed, and one faster speed). In other words, participants were tested executive function during four different workstation conditions. We hypothesized that the three components of executive function would not be influenced when participants were walking with a self-selected speed but would be impaired when participants were walking with a faster speed. We made these hypotheses based on the fact that walking was a highly automated skill, and thus walking with a self-selected speed should require minimum cognitive resources while walking with a faster speed may require increased cognitive resources. However, our results indicated there was no difference in executive function between walking with a self-selected and walking with a faster speed. In addition, we found that the use of active workstation had a selective impact on the three components of executive function. Detailed procedures of the experiment, results, and relevant discussions are presented next.

## Materials and methodsParticipants

Twenty-four college students (12 men, mean age = 24.0 years, SD = 1.5 years; 12 women, mean age = 22.1 years, SD = 1.5 years) were recruited by posted flyers. Potential participants were excluded if they were from Psychology Department, have color blindness or weakness, have neurological disorders, have a balance disorder, or have existing injuries that would restrict walking. All participants completed the Physical Activity Readiness Questionnaire (PAR-Q) [18] and provided written informed consent prior to the experiment. The research protocol was approved by the Institutional Review Board of Tsinghua University.

### Study design

The main independent variable of the current study was the workstation conditions, and the main dependent variable was the executive function performance, reflected by response time and accuracy. This study used a within-subject design, in which each participant performed a test battery of executive function under each of four conditions, including sitting, standing, walking at an active workstation with self-selected speed (self-paced walking), and walking at an active workstation with 1.5 times the self-selected speed (faster walking).

### Procedures

**Fig 1:**
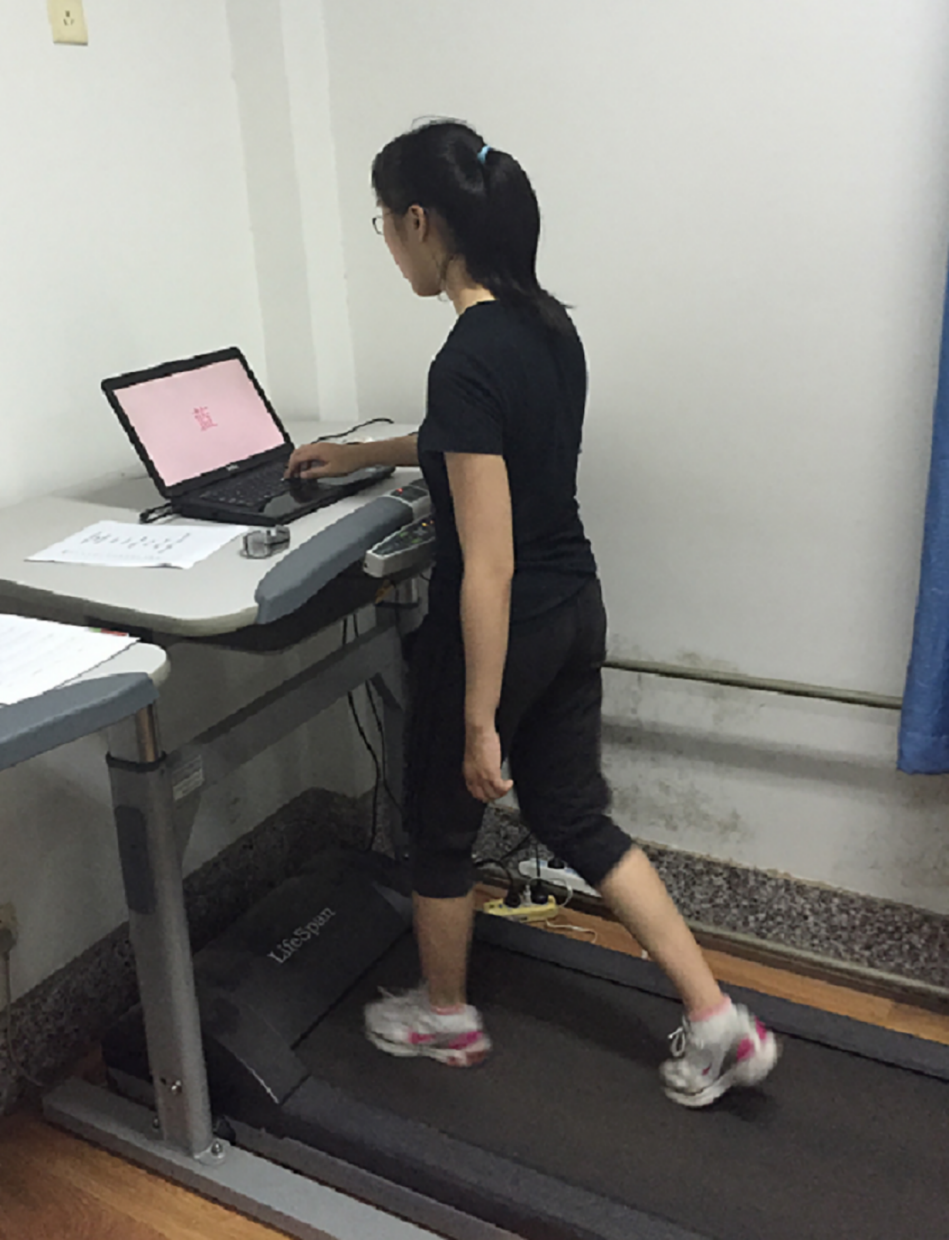
A participant was performing executive function test on an active workstation.

In April and May 2015, each participant visited the lab during four days with one day apart between two consecutive visiting days. In each day, participants performed the executive function test under one of the four experimental conditions (sitting, standing, self-paced walking, faster walking). In order to eliminate the order effects within the repeated measures design, the order in which participants performed the experimental conditions was counterbalanced (Table 1). During the sitting condition, participants performed the executive function test sitting at an office desk. During the standing condition and two walking conditions, participants performed the executive function test standing and walking on an active workstation, respectively (Fig 1). The active workstation used in this study (Lifespan, Salt Lake City, UT, USA) consisted of a treadmill and a height-adjustable desk. Prior to the first time using the active workstation, each participant had 10 minutes to practice walking on the active workstation. After participants got used to walking on the active workstation, self-selected speed was determined as their most comfortable walking speed on the active workstation.

**Table 1:** Sequence of test conditions.

Group	Day 1	Day 2	Day 3	Day 4
A	Sitting	Standing	Self-paced walking	Faster walking
B	Faster walking	Sitting	Standing	Self-paced walking
C	Self-paced walking	Faster walking	Sitting	Standing
D	Standing	Self-paced walking	Faster walking	Sitting

Note. Subjects were randomly divided into four groups (A, B, C, and D). Each row of the table showed the sequence of the test conditions of each group.

Participants visited the lab in the morning at two hours after breakfast and were not allowed to have exercised prior to the experiment that morning. When participants were performing the executive function test, a heart rate monitor (Polar Electro, Finland) was applied to identify the aerobic intensity of the active workstation condition. The executive function test started immediately after the participant reached a stable status of heart rate under each workstation condition. The entire test session lasted about 30 minutes.

### Measurements

The test battery of executive function consisted of three cognitive tasks to test the three components of the executive function respectively. There were 2-minute intervals between each task. The total duration of the executive function test was about 25 minutes. The program of the test battery was written using the Psychtoolbox package within Matlab (version 2014a).

#### Stroop task

Stroop Color Word task was used to measure the Inhibition function, in which participants were required to name a color word. There were congruent and incongruent for the trials. In the congruent trials, the name of the color word was same as the ink of the color word (e.g., the word “red” printed in red ink), while in the incongruent condition, the name of the color word was different as the ink of the color word (e.g., the word “red” printed in blue ink). In this study, there were six kinds of the trials, 1) the word “red” printed in red ink, 2)the word “blue” printed in blue ink, and 3) the word “green” printed in green ink, which were regarded as congruent condition, and 4) the word “red” printed in blue or green ink, 5) the word “blue” printed in red or green ink, and 6) the word “green” printed in red or blue ink, which were regarded as incongruent condition. There were 96 trials in which 48 trials were congruent and 48 trials were incongruent. Each stimulus was presented 2000ms and between two stimuli was 2 to 8 seconds interval with sign “+” presented on the screen. The stimuli were presented in a random order and participants were required to tell the color name of the words rather than the color of ink by pressing corresponding buttons on the keyboard.

#### N-back task

N-back task was used to measure the Updating function, which required participants to monitor a series of letters shown on the screen and match the current stimulus with the one that presented N steps earlier in the sequence. The parameter N in this study included 0, 1, 2, with increased number N associated with increased complexity of the task. In the 0-back task, the participants were only required to identify a pre-specified letter “X” (e.g., in a series letters “ABSXISXSD”, the underline letters were the target stimuli). In the 1-back task, the participants were required to identify the letter that was same as the last presented letter (e.g., in a series letters “ASXDDARRSIEE”, the underline letters were the target stimuli). In the 2-back task, the participants were required to identify the letter that was same as the letter presented prior to the last letter (e.g., in a series letters “ADFDJSJISISO”, the underline letters were the target stimuli). There were two blocks for each task and 18 trials in each block, in which 6 trials were target stimuli. Each stimulus was presented 500ms followed by 1500ms sign “+” presented at the center of the screen.

#### More-odd shifting task

More-odd shifting task was used to measure the Shifting function, which required participants to switch between different mental tasks and make corresponding responses. A series of number (1to 4, and 6 to 9) were presented at the center of the screen with two conditions: 1) if the color of the number was red, participants were required to identify whether the number is larger or smaller than 5 by pressing corresponding buttons on keyboard; and 2) if the color of the number was green, participants were required to identify whether the number is odd or even by pressing corresponding buttons on the keyboard. In the block “A”, all trials were red numbers, known as the “More-trials”. In the block “B”, all trials were green numbers, known as the “Odd-trials”. In the block “C”, trials were mixed conditions with red numbers and green numbers presented in a random order, known as the “Mixed-trials”. There were each 16 trials in block “A” and “B”, and 32 trials in block “C”. The order of the blocks was “ABCCBA” with each block presented twice. Each stimulus was presented 2000ms and between two stimuli was 3 seconds interval with sign “+” presented on the screen.

### Statistical analysis

In order to examine the effects of active workstation use on executive function, a series of analysis of variance (ANOVA) with repeated measures was conducted with workstation condition (sitting, standing, self-paced walking, faster walking) and trial type (congruence vs. incongruence for Stroop task; 0-, 1-, 2-back for N-back task; More-, Odd-, Mixed-trial for Shifting task) as within-subjects factors. Bonferroni’s post hoc procedure was used for post hoc comparisons if ANOVAs reported a significant main effect. Skewness and kurtosis of the data were checked for normality according to Kline’s criteria [19] prior to performing ANOVAs. The results indicated that most variables were normal except for the accuracy of 1-back task under sitting condition and the accuracy of congruent Stroop task under self-paced walking condition. Given that the ANOVA produces valid results even when the normality is violated [20], and given that the repeated measures design removes the individual differences, we tolerated the non-normality in these two variables, but nevertheless we considered it as a limitation. The main dependent variables in this study were the three components of executive function: Inhibition, Updating, and Shifting. The three components were determined by the average response time and accuracy of Stroop task, N-back task, and More-odd shifting task, respectively. Shorter response time and higher accuracy represented better performance. Statistical significant level for all analyses was set at p < 0.05. All data were collated and analyzed using IBM SPSS version 24.

## Results

### Heart rate

Table 1 presented the average heart rate of participants under different workstation conditions. The mean of the self-selected walking speeds on the active workstation was 2.3 km/h, ranging from 1.0 km/h to 3.1 km/h, and thus the average faster walking speed was 3.5 km/h. The average heart rate ranged from 74.9 beats per minute (bpm) in sitting condition to 99.4 bpm in faster walking condition. Repeated measures ANOVA showed a significant main effect for workstation conditions (F _(3, 69)_ = 77.814, p<0.001) on heart rate. Post-hoc comparisons revealed significant differences in heart rate between each two workstation conditions, with sitting (74.9 ±8.0 bpm) < standing (83.4 ± 12.2 bpm) < self-paced walking (93.4 ± 11.5 bpm) < faster walking (99.4 ±13.0 bpm, *p* values for all pairwise comparisons were < 0.05).

### Stroop task

**Fig 2:**
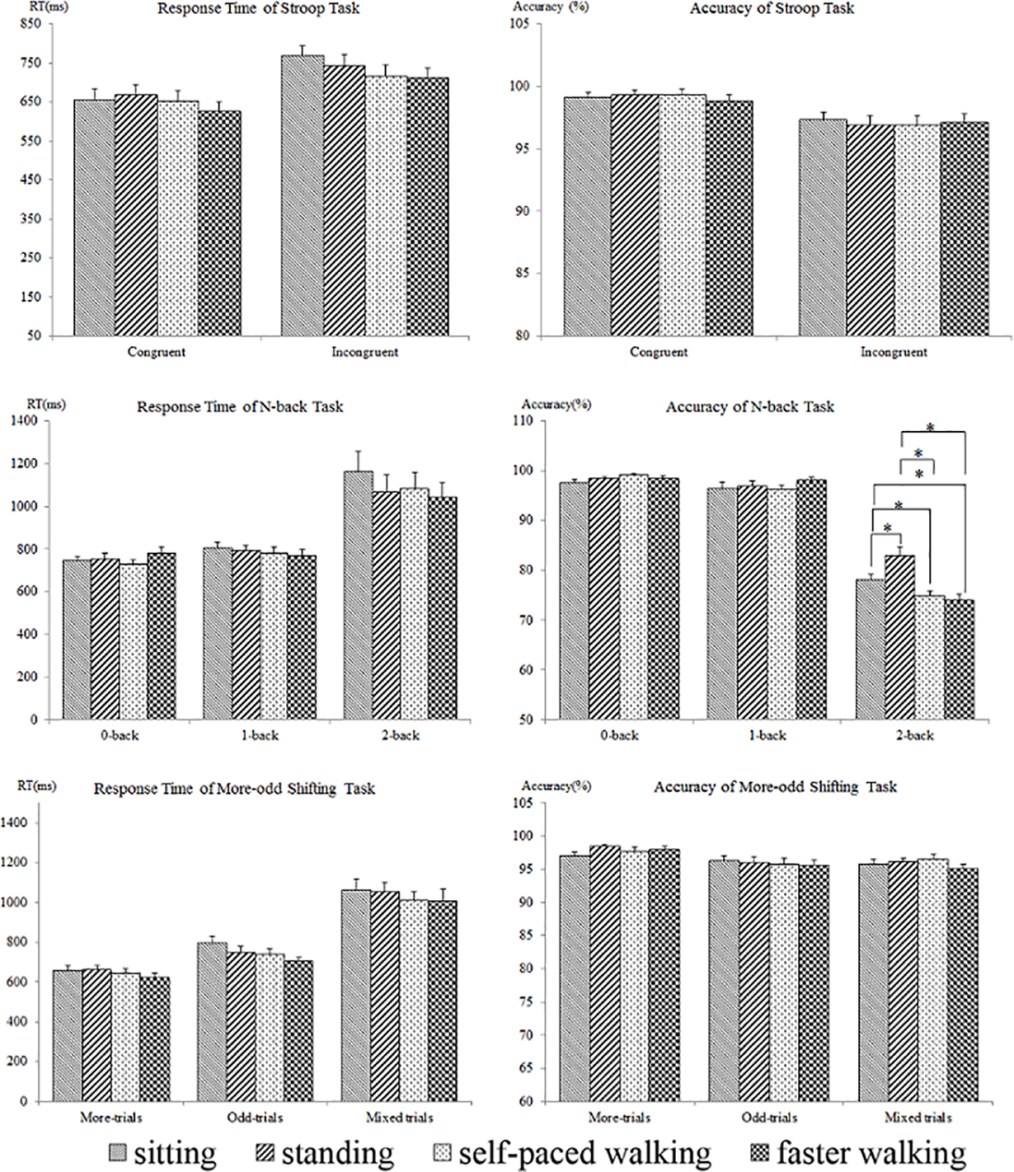
Comparisons of accuracy and response time among workstation conditions. Note: error bars denote standard error of the mean; asterisks denote significant pairwise comparisons.

Table 2 and Fig 2 showed the accuracy and the average response time of all executive function tests under four workstation conditions. For the Stroop task, there was a significant main effect for trial type in both accuracy (*F*_(1, 23)_ = 17.617, *p* < 0.001) and response time (*F*_(1, 23)_ = 55.373, *p* < 0.001), indicating that participants presented lower accuracy and longer response time in incongruent trials than in congruent trials. However, there was no significant main effect for workstation condition and no interaction between workstation condition × trial type in both accuracy and response time (all *p* values > 0.05), suggesting that the Stroop task performance did not vary across four workstation conditions.

**Table 2:** Accuracy and average response time of all executive function tests (mean ± SD).

Condition	Sitting	Standing	Self-paced walking	Faster walking
Stroop				
Accuracy (%)				
Congruent	99.1 ± 2.1	99.3 ± 1.9	99.3 ± 2.5	98.8 ± 2.3
Incongruent	97.3 ± 3.1	96.9 ± 3.7	96.9 ± 3.6	97.1 ± 3.5
Response time (ms)				
Congruent	654.1 ± 145.6	668.6 ± 123.2	652.1 ± 127.7	625.9 ± 117.7
Incongruent	768.0 ± 121.5	742.6 ± 137.4	716.2 ± 133.6	710.6 ± 125.9
N-back				
Accuracy (%)				
0-back	97.6 ± 3.8	98.4 ± 2.2	99.2 ± 1.5	98.5 ± 2.7
1-back	96.4 ± 6.9	96.9 ± 5.3	96.2 ± 4.2	98.1 ± 2.4
2-back	78.1 ± 5.5	83.0 ± 8.2	74.9 ± 4.5	74.0 ± 6.0
Response time (ms)				
0-back	743.2 ± 112.2	753.8 ± 144.3	728.1 ± 104.4	782.4 ± 140.2
1-back	804.4 ± 146.2	792.3 ± 120.3	781.1± 135.2	770.6 ± 125.1
2-back	1163.1 ± 635.7	1064.9 ± 400.7	1082.9 ± 369.0	1043.7 ± 325.2
More-odd shifting				
Accuracy (%)				
More-trials	97.0 ± 3.0	98.4 ± 1.8	97.7 ± 2.8	97.9 ± 2.6
Odd-trials	96.2 ± 4.1	96.0 ± 4.4	95.7 ± 4.4	95.6 ± 3.8
Mixed trials	95.8 ± 3.7	96.1 ± 2.8	96.5 ± 4.0	95.1 ± 3.1
Response time (ms)				
More-trials	658.4 ± 118.6	663.9 ± 94.3	646.2 ± 93.6	623.5 ± 94.0
Odd-trials	795.0 ± 172.5	749.7 ± 144.6	740.0 ± 129.4	703.1 ± 100.2
Mixed trials	1060.7 ± 278.4	1052.3 ± 233.9	1008.5 ± 213.4	1006.7 ± 288.6

### N-back task

Regarding the N-back task, there was a significant main effect for trial type in accuracy (*F*_(2, 46)_ = 677.319, *p* < 0.001). Post hoc comparisons indicated participants showed lower accuracy in 2-back task than in 0-back task (*p* < 0.001) and 1-back task (*p* < 0.001), but there was no difference in accuracy between 0-back task and 1-back task (*p* = 0.075). There was also a significant main effect for workstation condition in accuracy (*F*_(3, 69)_ = 4.029, *p* = 0.011) but it was superseded by interaction effect between workstation condition × trial type (*F*_(6, 138)_ = 9.371, *p* < 0.001). The decomposition of the interaction indicated the accuracy of 2-back task at self-paced walking (74.9%) was significantly lower than sitting (78.1%, *p* = 0.017) and standing (83.0%, *p* < 0.001). Accuracy of 2-back task at faster walking (74.0%) was also significantly lower than sitting (*p* < 0.015) and standing (*p* < 0.001). In addition, accuracy of 2-back task at sitting was significantly lower than standing (*p* = 0.017), but there was no significant difference between self-paced walking and faster walking (*p* = 0.517). No difference in accuracy across four workstation condition was found within the 0-back task and 1-back task.

There was also a significant main effect for trial type (*F*_(2, 46)_ = 26.963, *p* < 0.001) but no main effect for workstation condition (*p* = 0.739) and no interaction between workstation condition × trial type (*p* = 0.377) in response time. Post hoc comparisons indicated participants spent more time in 2-back task than in 1-back (*p* < 0.001) and 0-back task (*p* < 0.001). However, there was no difference in response time across four workstation condition within each task.

### More-odd shifting task

ANOVA revealed a significant main effect for trial type in both accuracy (*F*_(2, 46)_ = 9.912, *p* < 0.001) and response time (*F*_(2, 46)_ = 142.132, *p* < 0.001) of More-odd shifting task. Post hoc comparisons found participants showed lower accuracy and longer response time in Mixed-trials than that in More-trials and Odd-trials (all *p* values < 0.05). The accuracy in Odd-trials was significantly lower than in More-trials (*p* = 0.006), and the response time of Odd-trials was significantly longer than that of More-trials (*p* < 0.001). There was no significant main effect for workstation condition and no interaction between workstation condition × trial type in both accuracy and response time (all *p* values > 0.05), indicating the performance of More-odd shifting task did not differ across four workstation conditions.

## Discussion

According to a widely accepted theoretical model [17], this study tested the three core components of executive function under different workstation conditions. The results indicated that active workstation use had a selective impact on executive function.

Walking on the active workstation did not affect the Inhibition, a core component of the executive function, whether with a self-selected speed or with a faster speed. Inhibition was measured by Stroop Color Word task, in which participants were required to identify the color name of the word in two conditions, congruent or incongruent. For both congruent and incongruent trials, there was no significant difference in accuracy and response time across the four workstation condition (sitting, standing, self-paced walking, and faster walking). Our results were consistent with the findings by John et al. [21]and Alderman et al. [13], both of whom employed Stroop task as a measurement of selective attention. John et al. [21]found that walking at an active workstation at 1 mph did not affect the performance of Stroop compared to sitting. Alderman et al. [13]also found no significant difference in response speed and accuracy of Stroop task between walking and sitting conditions. The walking speed in Alderman et al.’s [13] study was self-selected and the average walking speed was 2.45km/h, which was very close to the average self-selected walking speed in the current study (2.3km/h). However, the current study found that walking with an even faster speed also did not influence the results of Stroop task compared to sitting.

Results in this study showed that the performance in both in 0-back and 1-back task did not differ across the four workstation conditions. But the performance in 2-back task was diminished during self-paced walking and faster walking compared to sitting, suggesting that the active workstation use might impair the Updating to a certain extent and such impairment was dependent on the working memory load. Updating, which is another component of the executive function, was assessed by N-back task, in which participants were required to match the current information with previously presented information, so the working memory was largely involved in the N-back task. The increase of the parameter N (from 0 to 2 in the current study) was associated with increasing complexity of N-back task because of the elevating working memory load, which could be indicated from the decreasing accuracy and increasing response time from 0-back task to 2-back task.

Shifting ability was not affected by the active workstation use. Shifting was evaluated by the More-odd shifting task, in which participants were required to quickly switch between two different tasks, one to judge the size of a number and the other to judge the odd-even of a number. This result is in line with Ehmann et al. study [14], which adopted Wisconsin Card Sorting test as a measure of cognitive flexibility and found the cognitive flexibility was unaffected during walking on an active workstation with self-preferred speed compared to sitting. Overall, our study found that active workstation use had a selective impact on the three components of the executive function. Active workstation use did not affect the Inhibition and Shifting but caused the impairment of Updating to some extent. Since the Updating component of executive function is highly related to working memory, our findings suggest that working memory might be more affected than other cognitive processes when using an active workstation. Previous studies regarding the effects of the use of active workstation on executive function mainly focused on the Inhibition component of the executive function [13, 21]. The current study extended the extant scholarship by examining the effects of the use of active workstation on the three components of executive function separately. Our findings showing a selective impact of the use of active workstation also indirectly support the Miyake et al.’s [17] proposition that the three components of executive function are distinguishable and should be assessed separately [13].

There might be two potential mechanisms to explain the change of cognitive function under active workstation condition. First, according to the arousal theory [22], the increased level of arousal induced by exercise intensity, which is usually measured by heart rate, oxygen uptake, or perceived exertion, has an inverted-U impact on the performance of cognitive tasks. The positive effect of arousal on cognitive function is also found to relate to the activation of central nervous system linked to the level of catecholamine [23]. A previous study indicated that the optimal zone of acute exercise intensity for cognitive performance ranged from 40% to 60% of maximal oxygen uptake [24]. Although the current study did not measure the oxygen uptake directly, we monitored the heart rate of participants during each workstation condition. It showed that the heart rate increased significantly from the sitting and standing conditions to active workstation conditions, indicating an elevating level of arousal. However, it is possible that such increase in arousal had not reached a level that was needed for altering the cognitive performance.

Second, under the active workstation condition, the cognitive tests were performed during low-intensity exercise, which could be regarded as a dual-task scenario. Participants were confronting one physical task and one cognitive task. According to the classic and temporary theories of attention, attention has a limited capacity and the cognitive performance might be compromised if there is competition for the attention resources [25]. Studies indicate that walking, although highly practiced, is not an entirely automated motor skill and need certain attention resources [26, 27]. Therefore, allocation of attention resources plays an important role in cognitive performance during active workstation condition. In addition, walking speed matters in the attention allocation when simultaneously performing a cognitive task and a physical task. Walking at a higher speed increases the instability of body and thus imposes a greater attentional demand on participants to maintain balance. A previous study found typing while walking at a lower speed (1.6 km/h) changed the gait kinematics and decreased the postural stability [28]. Funk et al. [29] compared the typing performance under four conditions including sitting, walking at 1.3 km/h, 2.25 km/h, and 3.2 km/h, and found that typing performance diminished when walking at 1.3 km/h and 3.2 km/h compared to sitting while there was no difference in typing performance between walking at 2.25 km/h and sitting, indicating there might exist an optimal zone of walking speed in active workstation use. Our study employed two different walking speeds including a self-selected speed and a faster speed which was 1.5 times the self-selected speed. However, we did not find any difference in cognitive test outcomes between two different walking speeds. It was possible that although the speeds in two walking conditions differed greatly, the exercise intensity did not differ much, which was indicated by the close heart rate under two active workstation conditions. It has been indicated that the attentional demand could be strongly related to the energy demand of the task, with greater energy demand associated with more attentional demand for controlling the movements [30]. Furthermore, the type of cognitive task could also impact the allocation of attention resources during dual-task scenario. Easier tasks might require fewer attention resources than more complex ones. It was supported in our study by the results of N-back task. While performing N-back task during walking, participants only showed decreased performance in 2-back task but not in 0-back task and 1-back task, in which the former one requires more working memory resources than the latter two.

### Limitations

Several study limitations should be noted. First, the study was limited to a narrow population of sample. The participants in our study were college students and not actually employed office workers, which might prevent the generalization of results to other population, such as middle age or elderly people, who might also be potential active workstation users. Second, we did not examine the role of physical fitness in the relationship between cognitive performance and active workstation use. It is possible that people with higher physical fitness level might feel more comfortable in using active workstation. A third limitation of the study was the short period of the assessment. It could be possible that prolonged use of active workstation might cause fatigue and thus impair cognitive function. In addition, effects of long-term use of active workstation on cognitive function were not examined in the current study. Long-term use might get people more accustomed to the active workstation and thus eliminate its potential negative effects. Future research is also suggested to investigate the delayed effects of active workstation use on cognitive functions as well as work performance.

Despite these limitations, our study has strengths in its counterbalanced design and testing different components of executive function. We employed self-selected walking speed rather than fixed walking speed for every participant since the preferred walking speed was known to differ among individuals based on various factors. In addition, the current study compared the effects of two different walking speeds in active workstation use on cognitive performance.

## Conclusions

In summary, the present study extended the extant knowledge on active workstation by examining the effects of active workstation use on the three components of executive function. The results showed that walking at an active workstation had a selective impact on the three components of executive function, in which Updating was impaired to a certain extent while Inhibition and Shifting were not affected. Since Updating is highly correlated to the working memory, it is indicated that active workstation use might be more compatible with non-working memory-intensive tasks. In conjunction with its ability to increase energy consumption and daily physical activity, which has been well demonstrated in previous studies, active workstation might be a feasible solution to eliminate sedentariness in work environment.

## Supporting information

S1 Dataset Table containing the raw data for all executive function tests. (CSV)
